# Lipoprotein(a) and Oxidized Phospholipids Promote Valve Calcification in Patients With Aortic Stenosis

**DOI:** 10.1016/j.jacc.2019.01.070

**Published:** 2019-05-07

**Authors:** Kang H. Zheng, Sotirios Tsimikas, Tania Pawade, Jeffrey Kroon, William S.A. Jenkins, Mhairi K. Doris, Audrey C. White, Nyanza K.L.M. Timmers, Jesper Hjortnaes, Maximillian A. Rogers, Elena Aikawa, Benoit J. Arsenault, Joseph L. Witztum, David E. Newby, Marlys L. Koschinsky, Zahi A. Fayad, Erik S.G. Stroes, S. Matthijs Boekholdt, Marc R. Dweck

**Affiliations:** a Department of Vascular Medicine, Academic Medical Center, Amsterdam UMC, Amsterdam, the Netherlands; b Division of Cardiovascular Medicine, Sulpizio Cardiovascular Center, University of California, San Diego, La Jolla, California; c British Heart Foundation Centre for Cardiovascular Science, University of Edinburgh, Edinburgh, United Kingdom; d Department of Cardiothoracic Surgery, UMC Utrecht, Utrecht, the Netherlands; e Cardiovascular Medicine, Brigham and Women’s Hospital, Harvard Medical School, Boston, Massachusetts; f Centre de recherche de l’Institut Universitaire de cardiologie et de pneumologie de Québec–Université Laval, Québec City, Québec, Canada; g Department of Medicine, Division of Endocrinology and Metabolism, University of California, San Diego, La Jolla, California; h Robarts Research Institute, University of Western Ontario, London, Ontario, Canada; i Translational and Molecular Imaging Institute, Icahn School of Medicine at Mount Sinai, New York, New York; j Department of Cardiology, Academic Medical Center, Amsterdam UMC, Amsterdam, the Netherlands

**Keywords:** aortic valve stenosis, calcific aortic valve disease, lipoprotein(a), oxidized phospholipids, valvular interstitial cells, ^18^F-NaF, fluorine-18 sodium fluoride, apoB, apolipoprotein B-100, AS, aortic valve stenosis, AVR, aortic valve replacement, CT, computed tomography, Lp(a), lipoprotein(a), OxPL, oxidized phospholipids, PET, positron emission tomography, TBR, tissue-to-background ratio, VIC, valvular interstitial cell

## Abstract

**Background:**

Lipoprotein(a) [Lp(a)], a major carrier of oxidized phospholipids (OxPL), is associated with an increased incidence of aortic stenosis (AS). However, it remains unclear whether elevated Lp(a) and OxPL drive disease progression and are therefore targets for therapeutic intervention.

**Objectives:**

This study investigated whether Lp(a) and OxPL on apolipoprotein B-100 (OxPL-apoB) levels are associated with disease activity, disease progression, and clinical events in AS patients, along with the mechanisms underlying any associations.

**Methods:**

This study combined 2 prospective cohorts and measured Lp(a) and OxPL-apoB levels in patients with AS (V_max_ >2.0 m/s), who underwent baseline ^18^F-sodium fluoride (^18^F-NaF) positron emission tomography (PET), repeat computed tomography calcium scoring, and repeat echocardiography. In vitro studies investigated the effects of Lp(a) and OxPL on valvular interstitial cells.

**Results:**

Overall, 145 patients were studied (68% men; age 70.3 ± 9.9 years). On baseline positron emission tomography, patients in the top Lp(a) tertile had increased valve calcification activity compared with those in lower tertiles (n = 79; ^18^F-NaF tissue-to-background ratio of the most diseased segment: 2.16 vs. 1.97; p = 0.043). During follow-up, patients in the top Lp(a) tertile had increased progression of valvular computed tomography calcium score (n = 51; 309 AU/year [interquartile range: 142 to 483 AU/year] vs. 93 AU/year [interquartile range: 56 to 296 AU/year; p = 0.015), faster hemodynamic progression on echocardiography (n = 129; 0.23 ± 0.20 m/s/year vs. 0.14 ± 0.20 m/s/year] p = 0.019), and increased risk for aortic valve replacement and death (n = 145; hazard ratio: 1.87; 95% CI: 1.13 to 3.08; p = 0.014), compared with lower tertiles. Similar results were noted with OxPL-apoB. In vitro, Lp(a) induced osteogenic differentiation of valvular interstitial cells, mediated by OxPL and inhibited with the E06 monoclonal antibody against OxPL.

**Conclusions:**

In patients with AS, Lp(a) and OxPL drive valve calcification and disease progression. These findings suggest lowering Lp(a) or inactivating OxPL may slow AS progression and provide a rationale for clinical trials to test this hypothesis.

Aortic valve stenosis (AS) is the commonest form of valvular heart disease in developed countries, and its disease burden is anticipated to double over the next 50 years (1). Following the failure of statins in reducing AS progression (2), effective medical therapies are lacking. The only treatment option is surgical or transcatheter aortic valve replacement (AVR), after patients develop severe stenosis and symptoms (3). Patients with AS are elderly, often have multiple comorbidities and are not well suited to major surgery or intervention. Moreover, these procedures are expensive and are associated with perioperative as well as long-term morbidity and mortality. There is, therefore, an unmet clinical need for novel therapies to slow AS progression, thereby obviating the requirement for AVR altogether.

Lipoprotein(a) (Lp[a]) is a major carrier of oxidized phospholipids (OxPL) 4, 5 and has been established as a causal risk factor for AS in several genetic and population studies 6, 7, 8. Lp(a) is unaffected by statin therapy, but levels can now be reduced with novel compounds (9), making Lp(a) a potential therapeutic target in AS. The risk factors associated with incident AS may differ from those associated with disease progression, due to pathophysiological differences in the initiation and propagation phases of the disease (10). Before Lp(a) or its associated OxPL can be considered viable targets for therapeutic intervention in AS, prospective longitudinal studies are required to establish whether Lp(a) and OxPL are associated with disease progression—in addition to mechanistic work investigating the cellular mechanisms linking Lp(a) and OxPL with valvular calcification.

In a prior study investigating patients with mild to moderate AS, elevated Lp(a) and OxPL on apolipoprotein B-100 (apoB) were associated with higher rates of disease progression determined by echocardiography, as well as an increased need for AVR (11). However, this finding has not been validated in the elderly patients encountered in routine clinical practice nor in patients with more advanced aortic stenosis. Moreover, the relationship of Lp(a) and OxPL to sensitive measures of aortic valve calcification such as computed tomography (CT) calcium scoring or ^18^F-sodium fluoride (NaF) positron emission tomography (PET) (a marker of calcification activity) has not been evaluated.

In this study, we analyzed a clinically representative cohort of patients with AS and compared baseline Lp(a) and OxPL-apoB levels to calcification activity in the aortic valve using ^18^F-NaF PET; to future AS progression, as assessed both by echocardiography and CT calcium scoring; and to future clinical events. In parallel, we performed mechanistic in vitro experiments to investigate the effects of Lp(a) and OxPL on valvular interstitial cell (VIC) calcification and whether these effects may be counteracted.

## Methods

### Study populations

**Figure 1:**
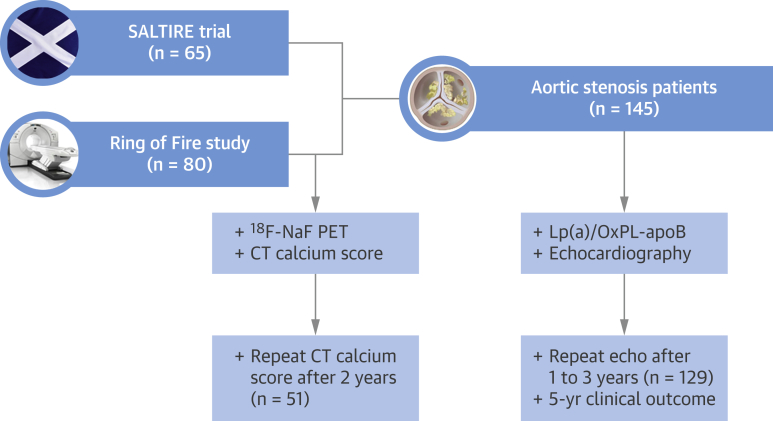
Study Diagram Study diagram of the study cohorts, describing the number of patients from the Ring of Fire and SALTIRE trials undergoing the different forms of baseline and repeat imaging. ^18^F-NaF PET = fluorine-18 sodium fluoride positron emission tomography; CT = computed tomography; Lp(a) = lipoprotein(a); OxPL-apoB = oxidized phospholipids on apolipoprotein B-100; SALTIRE = Scottish Aortic Stenosis and Lipid Lowering Trial, Impact on Regression.

Patients investigated in this study were drawn from across 2 multimodality imaging studies that prospectively collected data with respect to AS progression and adverse clinical events: the Ring of Fire study (12) and the SALTIRE (Scottish Aortic Stenosis and Lipid Lowering Trial, Impact on Regression) (13) (Figure 1). Both studies were conducted with local research ethics committee approval and with the written informed consent of all participants.

In the Ring of Fire study, patients age >50 years and matched control subjects were recruited from the outpatient department of the Edinburgh Heart Centre. PET, CT calcium scoring, and echocardiography were performed to assess disease activity and progression in a total of 81 patients. Patients were prospectively followed at the outpatient clinic for adverse clinical outcomes. For the present study, we included all AS patients who had baseline samples available for Lp(a) and OxPL measurements, as well as available progression data: 80 of 81 (98.8%) patients.

SALTIRE was a prospective trial assessing the effect of high-dose statin on AS progression. Patients with calcific AS, defined as a peak aortic jet velocity >2.5 m/s and aortic valve calcification on echocardiography, were eligible for inclusion. The main exclusion criteria were pre-existing statin therapy, planned aortic valve replacement, and moderate or severe left ventricular systolic impairment. Echocardiography was performed at baseline, at each annual visit, and before withdrawal from the trial. For the present study, all patients who had baseline Lp(a) and OxPL-apoB measurements and available echocardiographic progression data were included: 65 of 155 (41.9%) patients. These patients had similar baseline characteristics compared with the subjects in whom baseline Lp(a) and OxPL measurements were not available (Online Table 1).

### Lp(a), OxPL and autotaxin measurements

Plasma Lp(a) and OxPL-apoB were measured in all patients in both cohorts using chemiluminescent immunoassays, as previously described (11). Autotaxin (ATX) associated with Lp(a) and apoB was also measured (5).

### Baseline calcification activity with ^18^F-NaF PET

^18^F-NaF PET provides a measure of valvular calcification activity in aortic stenosis, binding preferentially to developing tissue microcalcification and predicting AS progression 12, 14, 15, 16, 17. In this study, aortic valve calcification activity was assessed at baseline in the Ring of Fire cohort using ^18^F-NaF PET/CT on a hybrid scanner (Biograph mCT, Siemens Medical Systems, Erlangen, Germany) 60 min after administration of 125 MBq of ^18^F-NaF.

PET data were reconstructed with the use of the Siemens Ultra-HD reconstruction algorithm. Corrections were applied for attenuation, dead time, scatter and random coincidences. Image analysis was performed on fused PET/CT datasets on a dedicated workstation (Osirix version 3.5.1. 64-bit, Osirix Imaging Software, Geneva, Switzerland). As previously reported, regions of interest were drawn around the aortic valve on reoriented coaxial PET/CT slices in the plane of the aortic valve, generating standard uptake values (SUV) (17). In a most diseased segment (MDS) approach, the 2 contiguous valve slices with the highest SUV values were averaged to generate the SUV_MDS_. These SUV values were corrected for blood-pool activity measured in the right atrium to generate tissue-to-background-ratio values (TBR_MDSmean_) (17).

### Progression in CT calcium scores

Progression in the aortic valve CT calcium score was assessed in patients from the Ring of Fire study. An ECG-gated breath-hold CT scan (noncontrast-enhanced, 40 mA/rot, 100 kV) was performed at baseline for calculation of the aortic valve calcium score on a hybrid PET/CT scanner (Biograph mCT). Repeat CT calcium scores were performed on the same scanner using the same protocol, and annualized change in CT calcium score calculated for each patient as previously reported (14). CT scans acquired in the SALTIRE trial were performed using an older scanner without ECG-gating and were therefore not deemed suitable for analysis.

### Hemodynamic progression on echocardiography

All 145 patients from both cohorts underwent assessment of hemodynamic AS severity and disease progression by the same research ultrasonographer (A.W.) on a dedicated echocardiography machine following a standardized protocol and according to 2006 American Heart Association/American College of Cardiology criteria (Online Appendix) 12, 13. Progression in AS disease severity was assessed by calculating the annualized change in peak transvalvular aortic valve velocity, using the final echocardiography data available collected at 1, 2, or 3 years after baseline assessments.

### Clinical outcomes

Clinical outcomes were collected prospectively across both patient cohorts and adjudicated by 2 independent and blinded investigators. The primary clinical endpoint was a composite of AVR and death, and the secondary endpoint was a composite of AVR and cardiovascular death. AVR was collected from individual electronic patient records. Deaths and cause of death were captured in the General Register of Scotland. All deaths were confirmed by independent review of each patient’s electronic health care record where available.

### Experimental methods

Expanded experimental methods and materials are presented in the Online Appendix. In brief, human VICs from control nonmineralized aortic valves were isolated and treated with TGF-β (positive control), Lp(a) isolated from healthy subjects (with or without monoclonal antibody E06), or recombinant apolipoprotein(a) constructs 17K-WT and 17KΔLBS10.

### Statistical analysis

This study is a post hoc analysis of 2 pooled cohorts in whom samples were available for Lp(a) and OxPL-apoB measurements; no formal sample size evaluations were therefore performed. However, post hoc power analyses were performed using nQuery Advisor version 7.0 (Statistical Solutions, Cork, Ireland) and are reported in the Online Appendix. Data are presented as mean ± SD for continuous variables with normal distribution, medians with interquartile ranges for continuous variables with non-normal distribution, and number (percentage) for categorical variables. Normality was assessed by inspection of histograms and the Shapiro-Wilk test. To assess differences in continuous data across Lp(a) and OxPL tertiles (top tertile vs. combined middle and bottom tertiles), an unpaired Student’s *t*-test or Mann-Whitney *U* test was performed, as appropriate. To assess differences in categorical data across Lp(a) and OxPL tertiles, a chi-square test was performed. In multiple regression analysis, dependent variables were ^18^F-NaF uptake, aortic valve calcium score, and progression of peak aortic jet velocity. Independent variables included the baseline peak aortic jet velocity, baseline aortic valve calcium score, Lp(a) and OxPL-apoB tertiles, and the traditional cardiovascular risk factor variables (age, sex, body mass index, history of cardiovascular disease, smoking status, diabetes mellitus, hypertension, and plasma creatinine). Following sensitivity analyses, baseline low-density lipoprotein cholesterol was not included in the multiple regression analysis (Online Table 2). Kaplan-Meier curves of time-to-event data were compared with the use of the log-rank test. Cox proportional hazards models were used to calculate hazard ratios by Lp(a) and OxPL-apoB tertiles. Unpaired Student’s *t*-tests were used to define differences between experimental conditions. Statistical analyses were performed using the SPSS statistics software version 24 (IBM, Armonk, New York). A p value <0.05 was considered statistically significant.

## Results

### Study cohort and baseline characteristics

**Figure 2:**
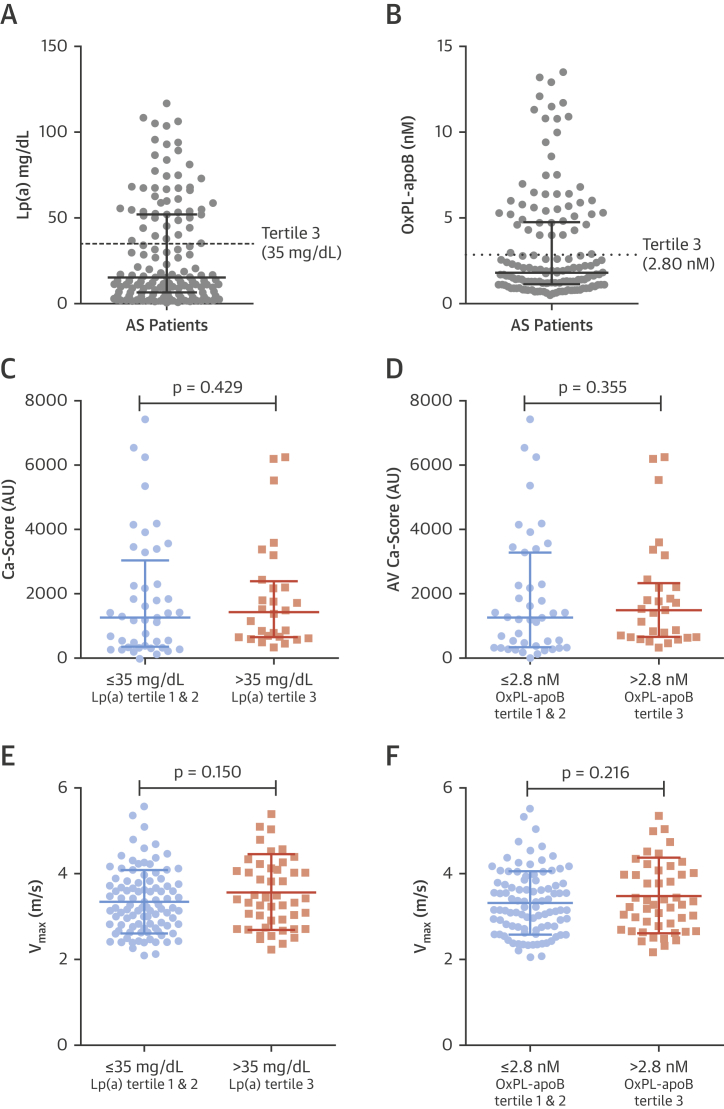
Baseline Lp(a) and OxPL-apoB Levels in Our Cohort and Association With Baseline Aortic Valve CT Calcium Score and Peak Aortic Valve Jet Velocity on Echocardiography **(A)** Lp(a) and **(B)** OxPL-apoB distribution in 145 patients. Baseline aortic valve CT calcium scores for the top versus combined middle and bottom tertiles of **(C)** Lp(a) and **(D)** OxPL-apoB. Baseline peak aortic jet velocity for the top versus combined middle and bottom tertiles of **(E)** Lp(a) and **(F)** OxPL-apoB. Abbreviations as in Figure 1.

After combining available data from the Ring of Fire cohort and the SALTIRE trial, a total of 145 elderly patients had samples available for Lp(a) and OxPL-apoB measurements (Figures 2A and 2B). In both studies, disease severity assessed by echocardiography was comparable (Online Table 3). The average age was 70.3 ± 9.9 years, and patients had a high prevalence of comorbidity. Baseline characteristics for the combined cohort are listed in Table 1, according to the top Lp(a) tertile (>35 mg/dl) versus combined middle and bottom tertiles (Lp(a) ≤35 mg/dl). Patients in the top Lp(a) tertile were well balanced compared with the lower tertiles with respect to baseline clinical characteristics, CT aortic valve calcium scores, and echocardiographic measures (Figures 2C and 2E). Similarly, baseline clinical and imaging characteristics were well balanced between patients in the top (>2.8 nmol/l) versus lower OxPL-apoB tertiles (≤2.8 nmol/l) (Figures 2D and 2F and Online Table 4).

**Table 1:** Baseline Characteristics of Patients

	All Patients (N = 145)	Lp(a) Levels		p Value
		Tertiles 1 and 2	Tertile 3	
		≤35 mg/dl (n = 96)	>35 mg/dl (n = 49)	
Clinical parameters				
Age, yrs	70.3 ± 9.9	69.5 ± 9.8	72.0 ± 10.0	0.14
Male	99 (68.3)	67 (69.8)	32 (65.3)	0.58
Body mass index, kg/m^2^	27.6 ± 4.3	27.6 ± 4.3	27.6 ± 4.5	0.94
Ischemic heart disease	37 (26.2)	22 (22.9)	15 (30.6)	0.31
Cardiovascular disease	53 (36.6)	32 (33.3)	21 (42.9)	0.26
Smoking (active or former)	92 (63.4)	60 (62.5)	32 (66.7)	0.62
Diabetes mellitus	15 (10.3)	8 (8.4)	8 (16.3)	0.15
Hypertension	83 (57.2)	55 (57.3)	28 (58.3)	0.91
Laboratory data				
Creatinine, mg/dl	91.8 ± 24.7	91.7 ± 22.6	92.1 ± 28.7	0.92
Urea, mg/dl	6.9 ± 2.5	6.74 ± 2.04	7.26 ± 3.18	0.24
Calcium, mg/dl	2.3 ± 0.1	2.32 ± 0.10	2.34 ± 0.18	0.27
Alkaline phosphatase, U/l	84.4 ± 41.2	83.3 ± 23.2	86.4 ± 62.9	0.74
Total cholesterol, mg/dl	206 ± 49	206 ± 50	207 ± 47	0.98
LDL cholesterol, mg/dl	111 ± 43	117 ± 43	100 ± 42	0.02
HDL cholesterol, mg/dl	56 ± 17	55 ± 17	58 ± 16	0.32
Triglycerides, mg/dl	133 (97–195)	137 (97–195)	133 (93–173)	0.53
Lipoprotein(a), mg/dl	15.2 (6.6–52.2)	9.5 (3.9–15.2)	61.1 (51.6–79.4)	—
OxPL-apoB, nmol/l	1.8 (1.2–4.8)	1.3 (0.9–1.8)	5.8 (4.7–9.0)	<0.001
Medication				
Statin at baseline	47 (32.4)	26 (27.1)	21 (42.9)	0.06
Statin during follow-up	82 (56.6)	50 (52.1)	32 (65.3)	0.13
ACE inhibitor	43 (29.7)	26 (27.1)	17 (34.4)	0.30
Echocardiography				
Peak aortic jet velocity, m/s	3.42 ± 0.79	3.36 ± 0.74	3.56 ± 0.89	0.15
Peak aortic valve gradient, mm Hg	48.9 ± 23.4	46.4 ± 20.5	53.8 ± 27.9	0.11
Mean aortic valve gradient, mm Hg	26.7 ± 14.2	25.5 ± 12.7	29.2 ± 16.6	0.14
Aortic valve area, cm^2^	1.07 ± 0.40	1.10 ± 0.42	1.02 ± 0.36	0.30

Values are mean ± SD, n (%), or median (interquartile range). LDL cholesterol was corrected for cholesterol content in Lp(a): LDL-C = LDL-C − Lp(a) mass × 0.3.

ACE = angiotensin-converting enzyme; HDL = high-density lipoprotein; LDL = low-density lipoprotein; Lp(a) = lipoprotein (a); OxPL-apoB = oxidized phospholipids on apolipoprotein B-100.

### Increased valvular calcification activity in patients with elevated Lp(a) and OxPL-apoB

**Figure 3:**
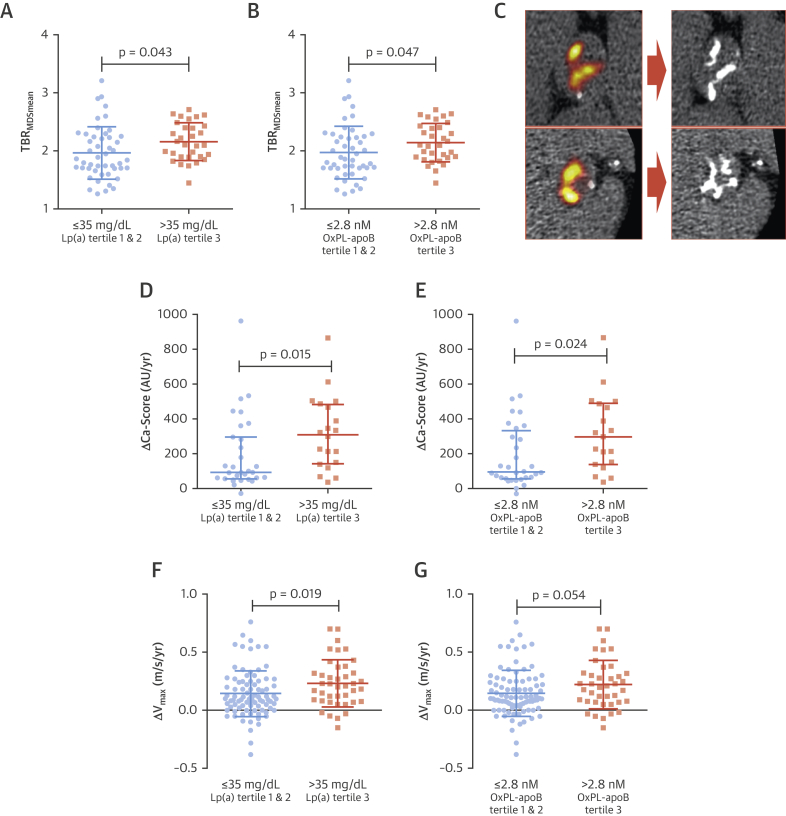
Baseline Calcification Activity and Aortic Stenosis Progression According to Patient Lp(a) and OxPL-apoB Levels At baseline, patients in the top Lp(a) and OxPL-apoB tertiles have increased valvular ^18^F-NaF uptake, as a marker of calcification activity, compared with the combined middle and bottom tertiles **(A and B)**. **(C)** Two examples of increased valvular ^18^F-NaF PET uptake at baseline **(left)**, which predicted progression to macrocalcification on CT after 2 years **(right)**(16). Patients in the top Lp(a) and OxPL-apoB tertiles also demonstrated faster progression in aortic valve CT calcium score **(D and E)**, and faster hemodynamic progression, expressed as the annualized change in peak aortic jet velocity (ΔV_max_) **(F and G)**. Abbreviations as in Figure 1.

To investigate calcification activity in the aortic valve, ^18^F-NaF PET/CT was performed in 80 patients from the Ring of Fire cohort (age 72.4 ± 8.4 years; 67% men). Patients in the top Lp(a) tertile demonstrated increased aortic valve ^18^F-NaF PET activity compared with patients in the lower Lp(a) tertiles (TBR_MDSmean_: 2.16 vs. 1.97; p = 0.043) (Figure 3A). Similarly, increased valvular ^18^F-NaF activity was observed in the top versus the lower OxPL-apoB tertiles (TBR_MDSmean_: 2.15 vs. 1.98; p = 0.047) (Figure 3B).

After adjustment for baseline calcium score and traditional cardiovascular risk factors, the top Lp(a) tertile was an independent predictor of ^18^F-NaF uptake (ß: 0.262 [95% CI: 0.095 to 0.410]; p = 0.002) as was the top OxPL-apoB tertile (ß: 0.204 [95% CI: 0.040 to 0.367]; p = 0.016) (Table 2, Online Table 5).

**Table 2:** Multiple Linear Regression Analysis

	^18^F-NaF PET Activity: TBR_MDSmean_			Progression in AV Calcium Score: ΔCa-Score (100 AU/yr)			Change in Peak Aortic Jet Velocity: V_max, m/s/yr_		
	Unst. β (95% CI)	Std. β	p Value	Unst. β (95% CI)	Std. β	p Value	Unst. β (95% CI)	Std. β	p Value
Age, per 10 yrs	0.100 (−0.019 to 0.218)	0.192	0.098	−0.253 (−1.016 to 0.511)	−0.082	0.507	0.006 (−0.034 to 0.046)	0.029	0.771
Male	0.160 (−0.087 to 0.408)	0.189	0.200	1.030 (−0.488 to 2.547)	0.225	0.178	−0.033 (−0.123 to 0.058)	−0.073	0.477
Body mass index, kg/m^2^	0.018 (−0.005 to 0.041)	0.179	0.126	−0.126 (−0.324 to 0.072)	−0.186	0.204	0.004 (−0.005 to 0.014)	0.092	0.349
Cardiovascular disease	−0.002 (−0.216 to 0.212)	−0.002	0.986	0.102 (−1.107 to 1.310)	0.023	0.866	0.029 (−0.057 to 0.114)	0.068	0.508
Smoking	−0.069 (−0.231 to 0.093)	−0.085	0.395	−0.559 (−1.505 to 0.386)	−0.128	0.238	−0.017 (−0.097 to 0.062)	−0.041	0.666
Diabetes mellitus	−0.164 (−0.422 to 0.094)	−0.151	0.208	0.056 (−1.662 to 1.774)	0.010	0.948	−0.086 (−0.222 to 0.050)	−0.124	0.214
Hypertension	0.048 (−0.145 to 0.241)	0.054	0.621	−0.406 (−1.561 to 0.749)	−0.084	0.481	−0.027 (−0.106 to 0.053)	−0.065	0.506
Creatinine, per 10 mg/dl	0.004 (−0.026 to 0.034)	0.026	0.811	−0.201 (−0.410 to 0.008)	−0.256	0.059	−0.001 (−0.018 to 0.015)	−0.016	0.874
Lipoprotein(a) top tertile	0.262 (0.095 to 0.410)	0.307	0.002	1.453 (0.515 to 2.391)	0.328	0.003	0.086 (0.007 to 0.166)	0.197	0.034
Baseline AV Ca-score, per 100 AU	0.012 (0.007 to 0.018)	0.529	<0.001	0.109 (0.071 to 0.147)	0.670	<0.001	—	—	—
Baseline V_max_, m/s	—	—	—	—	—	—	0.040 (−0.012 to 0.093)	0.144	0.130

AV Ca-score = aortic valve calcium score; CI = confidence interval; Std. β = standardized beta coefficient; TBR_MDSmean_ = tissue-to-background ratio of the most diseased segment; Unst. β = unstandardized beta coefficient; V_max_ = peak aortic jet velocity.

### Faster progression of CT calcium scores in patients with elevated Lp(a) and OxPL-apoB

Whether this increased calcification activity translated into faster progression of aortic valve calcium burden was subsequently assessed using repeat CT calcium scoring scans. A total of 51 patients underwent repeat CT calcium scoring at 2 years (age 72.6 ± 7.1 years; 66% men): the median progression in aortic valve calcium score was 3× faster for the top tertile of Lp(a) compared with the lower tertiles (309 AU/year [interquartile range: 142 to 483 AU/year] vs. 93 AU/year [interquartile range: 56 to 296 AU/year]; p = 0.015) (Figure 3D). Similarly, progression in aortic valve calcium score for the top OxPL-apoB tertile was 3× faster than in the lower tertiles (297 AU/year [interquartile range: 139 to 489 AU/year vs. 96 AU/year [interquartile range: 57 to 333 AU/year]; p = 0.024) (Figure 3E).

After adjustment for baseline calcium score and traditional cardiovascular risk factors, the top Lp(a) tertile was an independent predictor of annualized progression in the aortic valve calcium score (ß: 144 AU/year [95% CI: 52 to 239 AU/year]; p = 0.003) (Table 2). Similar results were observed for the top OxPL-apoB tertile, after the same multivariable adjustment (ß: 137 AU/year [95% CI: 43 to 230 AU/year]; p = 0.005) (Online Table 5).

### Faster hemodynamic progression in patients with elevated Lp(a) and OxPL-apoB

In total, repeat echocardiography was performed in 129 patients (89%), with final echocardiography data collected at 1 year in 11 (7.6%) patients, 2 years in 82 (56.6%) patients and 3 years in 36 (24.8%) patients. Annualized progression in hemodynamic AS severity as measured by the peak aortic jet velocity was almost double in the top versus the lower Lp(a) tertiles (0.23 ± 0.20 m/s/year vs. 0.14 ± 0.20 m/s/year; p = 0.019) (Figure 3F). Similar results were observed when comparing progression in the top versus lower OxPL-apoB tertiles (0.23 ± 0.21 m/s/year vs. 0.15 ± 0.20 m/s/year; p = 0.054) (Figure 3G).

In multiple regression analysis, the top Lp(a) tertile (ß: 0.086 m/s/year [95% CI: 0.007 to 0.166 m/s/year]; p = 0.034) was an independent predictor of progression in peak aortic jet velocity after adjusting for baseline peak aortic jet velocity and traditional cardiovascular risk factors (Table 2). Again, results were similar with respect to the top OxPL-apoB tertile (ß: 0.041 m/s/year [95% CI: −0.011 to 0.093 m/s/year]; p = 0.054) (Online Table 5).

### Higher event rates in patients with elevated Lp(a) and OxPL-apoB

**Figure 4:**
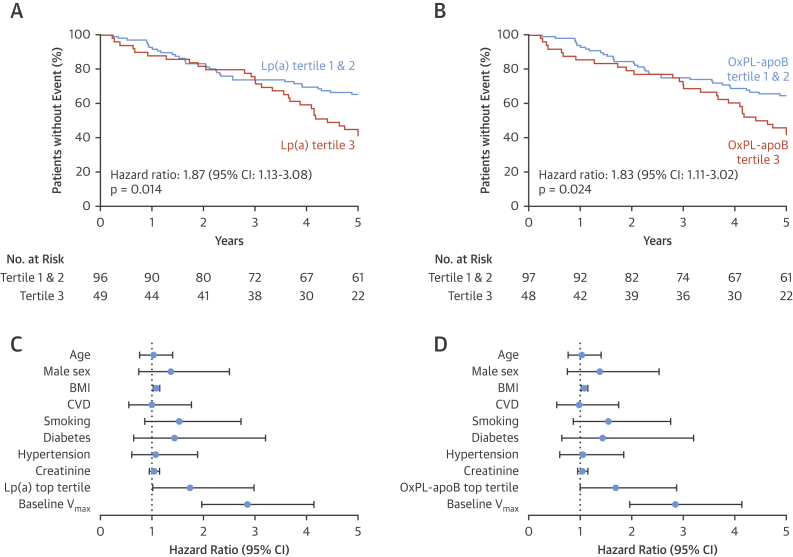
Aortic Valve Replacement or Death During Follow-Up According to Patient’s Lp(a) and OxPL-apoB Levels Kaplan-Meier curves show the composite endpoint of aortic valve replacement surgery and death. Patients in the top Lp(a) and OxPL-apoB tertiles had more events than those in the lower tertiles **(A and B)**. Multivariate Cox proportional hazards analysis for aortic valve replacement surgery and death. Lp(a) and OxPL-apoB emerged as an independent predictor of events alongside the baseline peak aortic jet velocity **(C and D)**. Hazard ratio for age is per 10 years, BMI per kg/m^2^, creatinine per 10 mg/dl; V_max_ per m/s. BMI = body mass index; CI = confidence interval; CVD = cardiovascular disease; V_max_ = peak aortic jet velocity; other abbreviations as in Figure 1.

After a median of 5 years follow-up (interquartile range: 2.7 to 5 years), 43 patients had undergone AVR and 22 patients had died, including 6 cardiovascular deaths and 3 patients who had AVR as their first event. The primary composite outcome of AVR or all-cause mortality occurred in 29 (59.2%) patients in the top tertile of Lp(a) and in 33 (34.4%) patients in the lower Lp(a) tertiles group (hazard ratio [HR] for Lp(a) top tertile: 1.87; 95% confidence interval [CI]: 1.13 to 3.08; p = 0.014) (Figure 4A). The primary composite endpoint occurred in 28 (58.3%) patients in the top OxPL-apoB tertile and 34 (35.1%) patients in the lower OxPL-apoB tertiles group (HR for OxPL-apoB top tertile: 1.83; 95% CI: 1.11 to 3.02; p = 0.028) (Figure 4B).

The secondary composite outcome of AVR or cardiovascular death occurred in 24 (49.0%) patients in the top tertile of Lp(a) and in 29 (30.2%) in the lower Lp(a) tertiles group (HR for Lp(a) top tertile: 1.72; 95% CI: 1.00 to 2.95; p = 0.050) (Online Figure 1A). The secondary composite endpoint occurred in 23 (47.9%) patients in the top tertile of OxPL-apoB and in 30 (30.9%) patients in the lower OxPL-apoB tertiles group (HR for top OxPL-apoB tertile: 1.68; 95% CI: 0.97 to 2.89; p = 0.063) (Online Figure 1B).

Adjustment for baseline peak aortic jet velocity and traditional cardiovascular risk factors did not markedly change the association of the primary composite endpoint, with both the top tertile of Lp(a) (adjusted HR: 1.74; 95% CI: 1.02 to 2.98; p = 0.044) (Figure 4C) and the top tertile of OxPL-apoB (adjusted HR: 1.69; 95% CI: 1.00 to 2.87; p = 0.050) (Figure 4D) emerging as independent predictors. Multivariable analyses for the secondary composite endpoint demonstrated comparable results (Online Figures 1C and D).

### Stratification by ATX reveals differences in hemodynamic progression

Complete results are presented in Online Table 6 and Online Figures 2 and 3. Patients with above-median levels of ATX-Lp(a) (0.20 ± 0.19 m/s/year vs. 0.09 ± 0.19 m/s/year; p = 0.012) and below median levels of ATX-apoB 0.10 ± 0.17 m/s/year vs. 0.19 ± 0.21 m/s/year; p = 0.028) had faster hemodynamic progression, but only in the lower tertiles of Lp(a) and OxPL-apoB, respectively (n = 87 in lower tertiles). There were no associations between ATX-Lp(a) or ATX-apoB and ^18^F-NaF uptake, progression in CT calcium score, or the composite outcome of AVR and death.

### Lp(a) induces osteogenic differentiation of VICs through its OxPL content

**Figure 5:**
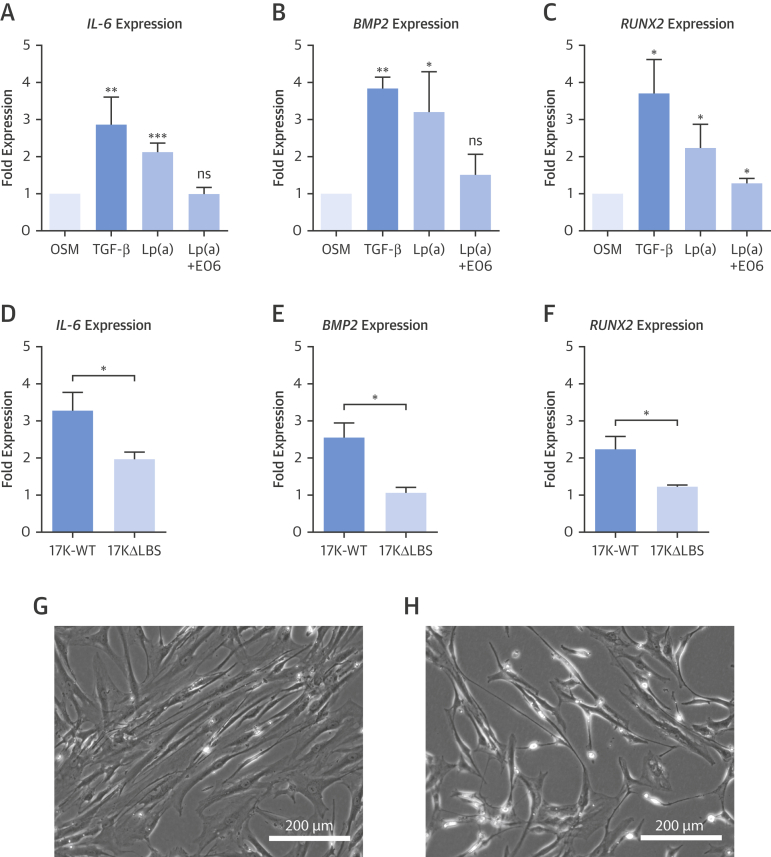
Lp(a) Induces Osteogenic Differentiation in VICs **(A to C)** VICs in osteogenic media only conditions were used as the baseline comparator, while addition of TGF-β served as a positive calcification control. One week of exposure to Lp(a) (100 mg/dl) induced gene expression of the inflammatory mediator *IL-6* and osteoblastic regulators *BMP2* and *RUNX2*. Pre-incubation with the E06 antibody targeting OxPL reduced the transcriptional effect of Lp(a), suggesting that OxPL is intrinsic to the pro-calcific effects of Lp(a). The role of OxPL was further validated using 2 r-apo(a) constructs that differ in their lysine binding sites and consequently their capacity to bind OxPL. **(D to F)** Three days of exposure to r-apo(a) 17K-WT construct induced increased expression of osteogenic genes. This effect was reduced with the 17KΔLBS10 construct that lacks the ability to bind OxPL. **(G and H)** Bright-field microscopy images of VICs after stimulation with 17K constructs. 17K-WT induces an activated, rhomboid shape, whereas 17KΔLBS10 was characterized by a quiescent, spindle-shaped morphology. Data represent mean ± SEM for at least n = 3 independent experiments. *p < 0.05; **p < 0.01; ***p < 0.001 compared with OSM only or comparison between 17K-WT and 17KΔLBS10. 17K-WT = 17 kringles wild-type r-apo(a) construct; 17KΔLBS = 17K r-apo(a) construct without lysine binding site; BMP2 = bone morphogenetic protein 2; E06 = E06 monoclonal antibody; IL-6 = interleukin-6; OSM = osteogenic medium; RUNX2 = Runt-related transcription factor 2; TGF-β = transforming growth factor beta. VIC = valvular interstitial cell.

To understand the mechanism by which Lp(a) might induce aortic valve calcification, we aimed to study the effect of Lp(a) on VICs. As a positive control for osteogenic differentiation of VICs, we used TGF-β, an established mediator of calcification of VICs in culture (18). First, we isolated Lp(a) from serum of healthy human subjects with elevated Lp(a) levels. After 1 week of exposure to Lp(a) 100 mg/dl, we evaluated osteogenic differentiation of VICs by assessing gene expression of the key inflammatory mediator *IL-6* and the major osteoblastic transcription factors *BMP2* and *RUNX2*. Lp(a) increased *IL-6* expression 2.1-fold (p = 0.009), *BMP2* expression 3.2-fold (p = 0.048) and *RUNX2* expression 2.2-fold (p = 0.020), compared with osteogenic medium only. Importantly, pre-incubation of Lp(a) with the E06 monoclonal antibody against OxPL markedly attenuated these Lp(a)-mediated osteogenic differentiation effects (Figures 5A to 5C).

Next, we addressed the role of the OxPL moiety on Lp(a) using 2 different r-apo(a) constructs, consisting of 17 kringles (17K) with different lysine binding sites (LBS) that affect their ability to bind OxPL covalently. The r-apo(a) 17K-WT construct contains an intact and functional LBS, while 17KΔLBS10 contains a mutation in the LBS that renders it defective in binding OxPL. Similar to Lp(a), the 17K-WT construct induced *IL-6*, *BMP-2,* and *RUNX2* expression. These transcriptional effects diminished with 17KΔLBS10, again supporting an important role for OxPL in mediating Lp(a)-induced VIC calcification (Figures 5D to 5F). In addition, when assessing cell morphology, 17K-WT induced an activated rhomboid shape, suggesting VIC activation or phenotype transformation. In contrast, VICs exposed to 17KΔLBS10 demonstrated a spindle-shaped morphology, corresponding to a quiescent state (Figures 5G and 5H).

## Discussion

In this multimodality imaging study, we present the novel finding that increased Lp(a) and OxPL-apoB levels in elderly patients with advanced AS are associated with increased valvular calcification activity using ^18^F-NaF PET and confirmed faster rates of disease progression using both CT calcium scoring and echocardiography (Central Illustration). This translated into an increased incidence of AVR and death. In vitro studies demonstrated that these observations appear to be driven by the pro-osteogenic effects of Lp(a) on VICs, mediated through its OxPL content, which could be alleviated with the E06 antibody that binds to and inactivates OxPL. Collectively, these data suggest that Lp(a) and its associated OxPL are important therapeutic targets in AS. Clinical trials are now warranted investigating whether novel Lp(a) lowering compounds or therapeutic antibodies targeting OxPL are effective in slowing disease progression in aortic stenosis.

**Central Illustration:**
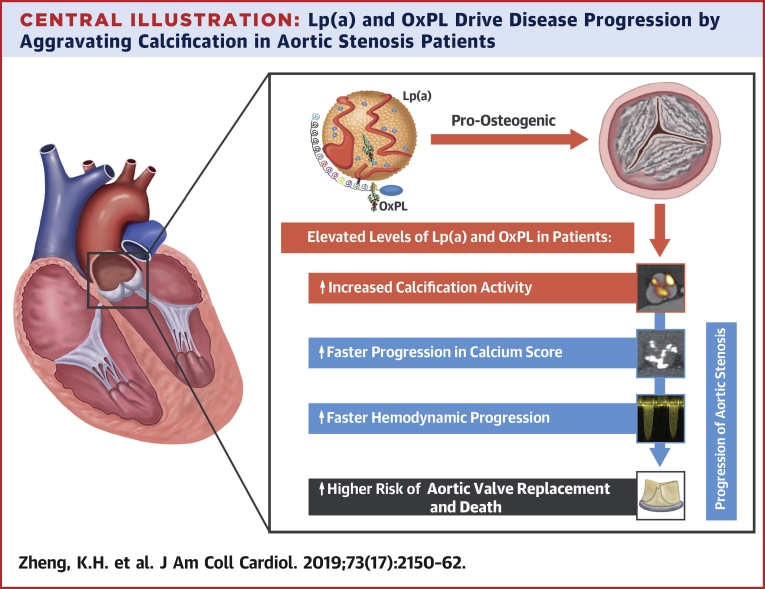
Lp(a) and OxPL Drive Disease Progression by Aggravating Calcification in Aortic Stenosis Patients Aortic stenosis patients with elevated levels of Lp(a) and OxPL-apoB demonstrate increased calcification activity in the valve as measured by ^18^F-NaF PET, compared with patients with low levels of Lp(a) and OxPL-apoB. During follow-up, this resulted in faster progression of CT calcium score and faster hemodynamic progression as measured by echocardiography. Ultimately, these patients have an increased risk of aortic valve replacement and death. Lp(a) = lipoprotein(a); OxPL-apoB = oxidized phospholipids on apolipoprotein B-100.

This is the first study to combine PET, CT, and echocardiography data to investigate the association among elevated Lp(a), OxPL, valve calcification activity, and disease progression in patients with AS. In patients with comparable disease severity at baseline, we demonstrate that elevated Lp(a) as well as OxPL-apoB levels are independently associated with increased valvular ^18^F-NaF uptake: a validated measure of active tissue calcification predicting AS progression (14). Indeed, this effect was reproduced when examining aortic stenosis progression directly, with elevated Lp(a) and OxPL-apoB independently associating with faster hemodynamic progression on echocardiography, faster progression in the aortic valve calcium score on CT, as well as an increased clinical event rate.

Our data support a post hoc analysis of the ASTRONOMER (Aortic Stenosis Progression Observation: Measuring Effects of Rosuvastatin) trial (11), in which patients with mild to moderate AS and elevated Lp(a) (top tertile >58.5 mg/dl) or OxPL-apoB levels were reported to have faster AS progression. However, the ASTRONOMER trial only examined echocardiographic progression. Furthermore, the patient population was considerably younger than that encountered in routine clinical practice (mean age 57 years), and nearly one-half of patients had a bicuspid valve. This led many to question the relevance of Lp(a) as a therapeutic target in the majority of elderly patients seen clinically with AS. Here, we find that in a study population with a mean age of 70 years, elevated Lp(a) (using a cut-off of 35 mg/dl) and OxPL-apoB (>2.8 nmol/l) were associated with a 2- to 3-fold faster rate of disease progression measured by echocardiography and CT calcium scoring. Our data therefore extend the generalizability of Lp(a) and its associated OxPL as independent risk factors driving AS progression to the wider clinical population and suggest that patients with elevated Lp(a) and OxPL-apoB levels might benefit from a reduction in these levels to slow disease progression.

What is the mechanism for these observations? Our in vitro data confirm that Lp(a) increases expression of key osteogenic genes in human VICs, committing them to an osteoblastic-like cell type (19). Using the monoclonal antibody E06, we demonstrate that blocking the OxPL moiety carried by Lp(a) diminishes the osteogenic response of VICs to Lp(a). Moreover, we substantiated the key role of OxPL in eliciting this pro-osteogenic response using r-apo(a) constructs with or without the capacity to bind OxPL. Together, these data support emerging published data that suggest an important role for the OxPL content of Lp(a) in valve calcification 5, 20, 21. They also suggest that novel therapies targeting OxPL may be useful in slowing aortic valvular calcification, as well as atherosclerosis, as recently demonstrated using a hypercholesterolemic mouse model expressing a single-chain variable fragment of E06 (22).

### Clinical implications

In the absence of any successful drug intervention able to reduce AS progression, it is instrumental to identify key factors contributing to this disease process. In line with epidemiological surveys and Mendelian randomization studies establishing the association of Lp(a) and OxPL with AS incidence (4), we have now confirmed that elevated Lp(a) and OxPL-apoB levels are associated with increased valve calcification activity and faster disease progression in a clinically representative cohort of AS patients. In view of the high prevalence of elevated Lp(a) levels in the general population (>50 mg/dl in up to 20%) (23), therapies aimed at lowering Lp(a) may have significant effect, particularly as they might also have beneficial effects on coexistent atherosclerotic disease. Alternatively, therapies seeking to inhibit valvular calcification may theoretically adversely affect any plaque-stabilizing effects of calcification in regions of atheroma. Although to our knowledge, no clinical data exist to support this hypothesis, future studies targeting calcification will need to be vigilant regarding their effects on atherosclerosis. Emerging therapies such as antisense oligonucleotides have shown that decreasing circulating Lp(a) levels by up to 95% is feasible with a favorable safety and tolerability profile (9). Randomized controlled trials will be needed to test the hypothesis that lowering Lp(a) or OxPL in patients with elevated levels truly slows down AS progression rates and ultimately improves clinical outcomes. Traditionally, such studies have concentrated on the effects of drugs on hemodynamic progression assessed by echocardiography. However, increasingly they are also examining progression in the CT calcium score and changes in the ^18^F-NaF PET signal (SALTIRE-2 [Study Investigating the Effect of Drugs Used to Treat Osteoporosis on the Progression of Calcific Aortic Stenosis], NCT02132026; BASIK2 [Bicuspid Aortic Valve Stenosis and the Effect of vItamin K2 on Calciummetabolism on 18F-NaF PET/MRI], NCT02917525), as used in the present study.

### Study limitations

We have pooled 2 prospective studies to increase power, but the number of patients per tertile remains relatively modest, conferring the risk of false positive findings. The Agatston method to quantify valvular calcification has inherent limitations that potentially decrease its accuracy in detecting disease progression (24). However, other methods for measuring valve calcium burden using CT remain relatively unexplored. Furthermore, there were differences in statin use across the cohorts, although randomized controlled trials and a recent meta-analysis demonstrated that statins do not impact hemodynamic disease progression or reduce valve-related events in AS (2). Finally, future studies with larger sample sizes are needed to improve our understanding of the Lp(a)-OxPL-ATX pathway in AS.

## Conclusions

In aortic stenosis, patients with elevated Lp(a) and OxPL-apoB plasma levels demonstrate increased valvular calcification activity, faster disease progression assessed both by echocardiography and CT, and an increased risk of AVR or death than subjects with lower levels. In vitro studies show that this effect appears to be mediated by the pro-osteogenic effects of Lp(a) and OxPL on valvular interstitial cells, and that these effects are potentially reversible with targeted treatment inactivating OxPL. These findings provide a rationale for clinical studies aiming to reduce elevated Lp(a) and OxPL levels in patients with aortic stenosis as a means of slowing disease progression and delaying the need for AVR.

Perspectives **COMPETENCY IN MEDICAL KNOWLEDGE:** Elevated blood levels of Lp(a) and OxPL-apoB are associated with accelerated valve calcification and hemodynamic progression of aortic stenosis.

**TRANSLATIONAL OUTLOOK:** Randomized trials are required to establish whether lowering Lp(a) and/or inhibiting OxPL can slow progression of clinical aortic stenosis.
