# Supplementary material for: Lipoprotein(a) and Oxidized Phospholipids Promote Valve Calcification in Patients With Aortic Stenosis
Source: J Am Coll Cardiol. 2019 May 7;73(17):2150–62. doi: 10.1016/j.jacc.2019.01.070 (PMC6494952; doi:10.1016/j.jacc.2019.01.070)
Supplement: Online Data [file mmc1.docx]

**Supplementary Material**

**Lipoprotein(a) and Oxidized Phospholipids Promote Valve Calcification in Patients with Aortic Stenosis**

Kang H. Zheng, MD,^a^ Sotirios Tsimikas, MD,^b^ Tania Pawade, MD, PhD,^c^ Jeffrey Kroon, PhD,^a^ William S.A. Jenkins, MD, PhD,^c^ Mhairi K. Doris, MD,^c^ Audrey C. White,^c^ Nyanza K.L.M. Timmers, ^a^ MD, Jesper Hjortnaes, MD, PhD,^d^ Maximillian A. Rogers, PhD,^e^ Elena Aikawa, MD, PhD,^e^ Benoit J. Arsenault, PhD,^f^ Joseph L. Witztum, MD,^g^ David E. Newby, MD, PhD,^c^ Marlys L. Koschinsky, PhD,^h^ Zahi A. Fayad, PhD,^i^ Erik S.G. Stroes, MD, PhD,^a^ S. Matthijs Boekholdt, MD, PhD,^j^ Marc R. Dweck, MD, PhD^c^

Affiliations: ^a^ Department of Vascular Medicine, Academic Medical Center, Amsterdam UMC, the Netherlands; ^b^ Division of Cardiovascular Medicine, Sulpizio Cardiovascular Center, University of California, San Diego, La Jolla, CA; ^c^ British Heart Foundation Centre for Cardiovascular Science, University of Edinburgh, United Kingdom; ^d^ Department of Cardiothoracic Surgery, UMC Utrecht, the Netherlands; ^e^ Cardiovascular Medicine, Brigham and Women’s Hospital, Harvard Medical School, Boston; ^f^ Centre de recherche de l’Institut Universitaire de cardiologie et de pneumologie de Québec, Université Laval, Canada; ^g^ Department of Medicine, Division of Endocrinology and Metabolism, University of California, San Diego, La Jolla, CA; ^h^ Robarts Research Institute, University of Western Ontario, Canada; ^i^ Translational and Molecular Imaging Institute, Icahn School of Medicine at Mount Sinai, New York; ^j^ Department of Cardiology, Academic Medical Center, Amsterdam UMC, the Netherlands

**Corresponding author:**

Dr. Marc R. Dweck

British Heart Foundation Centre for Cardiovascular Sciences

University of Edinburgh, The Chancellor’s Building

Little France Crescent, Edinburgh

Midlothian EH26 0NL

United Kingdom

Telephone: 0044 7813 619 208

Fax: 0044 131 242 6379

E-Mail: [marc.dweck@ed.ac.uk](mailto:marc.dweck@ed.ac.uk)

**Supplemental Methods**

*Echocardiography*

Patients were studied with the use of a 3-MHz or S51 pure wave transducer for 2-dimensional, M-mode and pulsed and continuous-wave Doppler scanning. All measurements were performed online and averaged from 3 cardiac cycles or 5 if the patient was in atrial fibrillation. AS severity was graded using the peak transvalvular aortic valve velocity, mean aortic valve pressure gradients, and aortic valve area according to the 2006 AHA/ACC criteria.(1)

*Cell culture*

Human VICs were isolated from control non-mineralized aortic valves obtained from patients undergoing heart transplantation.(2) Cells were cultured and expanded in SMC Growth Medium 2 (Promocell, Heidelberg, Germany) in non-coated, tissue-culture treated culture dishes. Cells between passage 4 and 7 were used for all experiments.

*Lp(a) and recombinant apolipoprotein(a) constructs*

The Lp(a) used in this study was isolated from serum from healthy subjects with elevated Lp(a) levels (> 50 mg/dL) by KBr-density gradient ultracentrifugation as previously described.(3) In brief, the gradient was built by layering consecutively 2 ml KBr solution (d=1.225 g/mL), 4 ml KBr solution (d=1.100 g/mL) and 3 ml KBr solution (d=1.006 g/mL) on top of the serum. Ultracentrifugation was run at 29.000 RPM for 19 hours at 10°C in an Optima XPN-100 ultracentrifuge system (Beckman, Fullerton, CA, USA). Lp(a) was obtained by tube slicing. Lp(a) concentrations were measured using the Architect CI8200 (Abbott Laboratories, Illinois). Recombinant apolipoprotein(a) (r-apo(a)) variants 17K wild-type (WT) and 17KΔLBS10 were generated as previously described.(4)

*Osteogenic stimulation*

VICs were cultured for 3 days or 1 week in osteogenic medium (DMEM containing 4.5 g/L glucose, L-glutamine, 5% FBS, 1% penicillin-streptomycin, 2 mmol/L NaH2PO4 and 50 µg/mL L-ascorbic acid). The medium was supplemented with one of the following stimuli: transforming growth factor beta as a positive control (TGF-β; R&D Systems, Minneapolis, 5 ng/ml); Lp(a) (100 mg/dL) with or without the E06 monoclonal antibody targeting OxPL (Avanti Polar Lipids, Alabama, 30 µg/mL); and the r-apo(a) constructs 17K-WT or 17KΔLBS10 (100 µg/mL for both).

*RNA isolation and quantitative PCR analysis*

RNA was extracted from cultured cells using Trizol and cDNA was synthesized with iScript (Biorad, Veenendaal, the Netherlands). qPCR was performed with 500 ng cDNA using SYBR Green Fast on a ViiA7 PCR machine (Applied Biosystems, Bleiswijk, the Netherlands). Primers for *BMP2*, *RUNX2*, *αSMA*, *IL6* and *36B4* were obtained from Sigma-Aldrich (Zwijndrecht, The Netherlands). Gene expression was normalized to the housekeeping gene *36B4*.

**Supplemental Table 1. Baseline characteristics for patients from SALTIRE, stratified by whether they had samples available for Lp(a) and OxPL-apoB testing.**

|  |  | **Sample available for Lp(a)/OxPL-apoB** | |  |
| --- | --- | --- | --- | --- |
|  | **All patients** | *No* | *Yes* | **p-value** |
| *n* | 155 | 90 | 65 |  |
| ***Clinical parameters*** |  |  |  |  |
| Age, years | 67.7 ± 10.6 | 67.5 ± 10.3 | 67.9 ± 11.1 | 0.82 |
| Male sex | 109 (70.3) | 64 (71.1) | 45 (69.2) | 0.80 |
| Body mass index, kg/m2 | 27.9 ± 4.6 | 28.3 ± 4.7 | 27.3 ± 4.5 | 0.18 |
| Ischemic heart disease | 30 (19.4) | 20 (22.2) | 10 (15.4) | 0.29 |
| Cardiovascular disease | 54 (34.8) | 32 (35.6) | 22 (33.8) | 0.83 |
| Smoking (active or former) | 100 (64.5) | 50 (55.6) | 50 (76.9) | 0.006 |
| Diabetes mellitus | 5 (3.2) | 3 (3.3) | 2 (3.1) | 0.93 |
| Hypertension | 79 (51.0) | 49 (54.4) | 30 (46.2) | 0.31 |
| ***Laboratory data*** |  |  |  |  |
| Creatinine, mg/dL | 91.7 ± 21.4 | 91.4 ± 23.2 | 92.1 ± 18.6 | 0.84 |
| Urea, mg/dL | 6.5 ± 5.0 | 6.8 ± 6.4 | 6.1 ± 1.9 | 0.36 |
| Calcium, mg/dL | 2.3 ± 0.1 | 2.3 ± 0.10 | 2.3 ± 0.10 | 0.06 |
| Alkaline phosphatase, U/L | 77.3 ± 24.0 | 74.0 ± 22.8 | 81.9 ± 25.1 | 0.04 |
| Total cholesterol, mg/dL | 221 ± 38 | 218 ± 39 | 224 ± 37 | 0.32 |
| LDL cholesterol, mg/dL | 136 ± 33 | 135 ± 34 | 137 ± 31 | 0.68 |
| HDL cholesterol, mg/dL | 58 ± 17 | 57 ± 16 | 62 ± 18 | 0.32 |
| Triglycerides, mg/dL | 115 (89-151) | 115 (89-151) | 124 (89-158) | 0.57 |
| ***Medication*** |  |  |  |  |
| Statin during follow-up | 77 (49.7) | 42 (46.7) | 35 (53.8) | 0.38 |
| ACE inhibitor | 20 (12.9) | 9 (10.0) | 11 (16.9) | 0.21 |
| ***Doppler echocardiography*** |  |  |  |  |
| Peak aortic jet velocity, m/s | 3.43 ± 0.64 | 3.49 ± 0.64 | 3.34 ± 0.63 | 0.22 |
| Peak aortic valve gradient, mmHg | 48.6 ± 18.4 | 50.2 ± 18.7 | 46.4 ± 17.9 | 0.20 |
| Mean aortic valve gradient, mmHg | 26.4 ± 10.9 | 27.5 ± 11.1 | 25.0 ± 10.6 | 0.14 |
| Aortic valve area, cm2 | 1.03 ± 0.41 | 1.08 ± 0.43 | 0.96 ± 0.36 | 0.08 |

Data are presented as mean ± standard deviation, median (interquartile range, or number (percentage). LDL cholesterol was corrected for cholesterol content in Lp(a): LDL-c = LDL-c – Lp(a) mass * 0.3. OxPL-apoB = oxidized phospholipids on apolipoprotein B-100.

**Supplemental Table 2. Sensitivity Analyses for LDL cholesterol**

| **Multiple linear regression model** | **18F-NaF PET activity: TBR_MDSmean_** | | | | **Progression in AV Ca-score: ∆Ca-score (100 AU/yr)** | | | | **Change in peak aortic jet velocity: V_max, (_m/s/yr)** | | | |
| --- | --- | --- | --- | --- | --- | --- | --- | --- | --- | --- | --- | --- |
| **Variable** | **Unstd. β (95% CI)** | **Std. β** | **p-value** | **Unst. β (95% CI)** | | **Std. β** | **p-value** | **Unstd. β (95% CI)** | | **Std. β** | **p-value** |  |
| Lp(a) top tertile | 0.193 (0.025 to 0.362) | 0.084 | 0.025 | 1.218 (0.189 to 2.066) | | 0.256 | 0.020 | 0.079 (0.003 to 0.157) | | 0.182 | 0.046 |  |
| LDL cholesterol, per 100 mg/dL | -0.062 (-0.244 to 0.121) | -0.066 | 0.504 | -0.311 (-1.472 to 0.850) | | -0.059 | 0.593 | -0.003 (-0.087 to 0.080) | | -0.007 | 0.936 |  |
| Baseline AV Ca-score, per 100 AU | 0.014 (0.009 to 0.019) | 0.570 | <0.001 | 0.103 (0.068 to 0.139) | | 0.638 | <0.001 | - | | - | - |  |
| Baseline V_max_, m/s | - | - | - | - | | - | - | 0.043 (-0.004 to 0.090) | | 0.160 | 0.072 |  |

| **Cox proportional hazards model** | **Composite endpoint AVR and death** | |
| --- | --- | --- |
| **Variable** | **Hazard ratio (95% CI)** | **p-value** |
| Lp(a) top tertile | 1.764 (1.055 to 2.951) | 0.031 |
| LDL cholesterol, per 100 mg/dL | 0.997 (0.991 to 1.004) | 0.421 |

AV Ca-score = aortic valve calcium score; AU = Agatston Units; V_max_ = peak aortic jet velocity; TBR_MDSmean_ = tissue-to-background ratio of the most diseased segment; Unst. β = unstandardized beta coefficient; Std. β = standardized beta coefficient; CI = confidence interval

**Supplemental Table 3. Baseline characteristics for included patients from SALTIRE and the Ring of Fire study**

|  |  | **Study cohort** | |  |
| --- | --- | --- | --- | --- |
|  | **All patients** | *SALTIRE* | *Ring of Fire* | **p-value** |
| *n* | 145 | 65 | 80 |  |
| ***Clinical parameters*** |  |  |  |  |
| Age, years | 70.3 ± 9.9 | 67.9 ± 11.1 | 72.3 ± 11.1 | 0.009 |
| Male sex | 99 (68.3) | 45 (69.2) | 54 (67.5) | 0.824 |
| Body mass index, kg/m2 | 27.6 ± 4.3 | 27.3 ± 4.5 | 27.9 ± 4.2 | 0.433 |
| Ischemic heart disease | 38 (26.2) | 10 (15.4) | 28 (35.0) | 0.008 |
| Cardiovascular disease | 53 (36.6) | 22 (33.8) | 31 (38.8) | 0.542 |
| Smoking (active or former) | 92 (63.4) | 50 (76.9) | 42 (52.5) | 0.002 |
| Diabetes mellitus | 15 (10.3) | 2 (3.1) | 13 (16.5) | 0.009 |
| Hypertension | 83 (57.2) | 30 (46.2) | 53 (66.3) | 0.015 |
| ***Laboratory data*** |  |  |  |  |
| Creatinine, mg/dL | 91.8 ± 24.7 | 92.1 ± 18.6 | 91.6 ± 28.9 | 0.902 |
| Urea, mg/dL | 6.9 ± 2.5 | 6.1 ± 1.9 | 7.6 ± 2.7 | <0.001 |
| Calcium, mg/dL | 2.32 ± 0.13 | 2.31 ± 0.10 | 2.34 ± 0.15 | 0.134 |
| Alkaline phosphatase, U/L | 84.4 ± 41.2 | 81.9 ± 25.1 | 86.4 ± 50.8 | 0.519 |
| Total cholesterol, mg/dL | 206 ± 49 | 224 ± 37 | 192 ± 52 | <0.001 |
| LDL cholesterol, mg/dL | 111 ± 43 | 130 ± 32 | 96 ± 45 | <0.001 |
| HDL cholesterol, mg/dL | 56 ± 17 | 62 ± 18 | 52 ± 15 | 0.002 |
| Triglycerides, mg/dL | 133 (97-195) | 124 (89-158) | 168 (106-210) | 0.007 |
| Lipoprotein(a), mg/dL | 15.2 (6.6-52.2) | 11.8 (5.1-37.4) | 17.5 (8.4-62.2) | 0.037 |
| OxPL-apoB, nM | 1.8 (1.2-4.8) | 1.5 (0.9-3.5) | 2.1 (1.3-5.5) | 0.012 |
| ***Medication*** |  |  |  |  |
| Statin during follow-up | 82 (56.6) | 35 (53.8) | 47 (58.8) | 0.554 |
| ACE inhibitor | 43 (29.7) | 11 (17.2) | 32 (40.0) | 0.003 |
| ***Echocardiography*** |  |  |  |  |
| Peak aortic jet velocity, m/s | 3.42 ± 0.79 | 3.34 ± 0.63 | 3.49 ± 0.90 | 0.282 |
| Peak aortic valve gradient, mmHg | 48.9 ± 23.4 | 46.4 ± 17.9 | 50.9 ± 27.1 | 0.247 |
| Mean aortic valve gradient, mmHg | 26.7 ± 14.2 | 25.0 ± 10.6 | 28.2 ± 16.4 | 0.173 |
| Aortic valve area, cm2 | 1.07 ± 0.40 | 0.96 ± 0.36 | 1.16 ± 0.41 | 0.002 |

Data are presented as mean ± standard deviation, median (interquartile range, or number (percentage). LDL cholesterol was corrected for cholesterol content in Lp(a): LDL-c = LDL-c – Lp(a) mass * 0.3. OxPL-apoB = oxidized phospholipids on apolipoprotein B-100.

**Supplemental Table 4. Baseline characteristics of patients stratified by OxPL tertiles**

|  |  | **OxPL-apoB levels** | |  |
| --- | --- | --- | --- | --- |
|  |  | Tertile 1 & 2 | Tertile 3 |  |
|  | **All patients** | *≤ 2.8 nM* | *> 2.8 nM* | **p-value** |
| *n* | 145 | 97 | 48 |  |
| ***Clinical parameters*** |  |  |  |  |
| Age, years | 70.3 ± 9.9 | 69.5 ± 9.8 | 72.0 ± 10.1 | 0.15 |
| Male sex | 99 (68.3) | 69 (71.1) | 30 (62.5) | 0.29 |
| Body mass index, kg/m2 | 27.6 ± 4.3 | 27.6 ± 4.2 | 27.7 ± 4.5 | 0.87 |
| Ischemic heart disease | 37 (25.5) | 22 (22.7) | 15 (31.3) | 0.27 |
| Cardiovascular disease | 52 (35.9) | 32 (33.0) | 20 (41.7) | 0.31 |
| Smoking (active or former) | 92 (63.4) | 62 (63.9) | 30 (62.5) | 0.87 |
| Diabetes mellitus | 16 (11.1) | 8 (8.3) | 8 (16.7) | 0.14 |
| Hypertension | 83 (57.2) | 54 (55.7) | 29 (60.4) | 0.59 |
| ***Laboratory data*** |  |  |  |  |
| Creatinine, mg/dL | 91.8 ± 24.7 | 91.6 ± 22.6 | 92.4 ± 28.8 | 0.86 |
| Urea, mg/dL | 6.9 ± 2.5 | 6.71 ± 2.06 | 7.32 ± 3.17 | 0.17 |
| Calcium, mg/dL | 2.32 ± 0.13 | 2.31 ± 0.10 | 2.34 ± 0.18 | 0.24 |
| Alkaline phosphatase, U/L | 84.4 ± 41.2 | 83.6 ± 23.2 | 85.9 ± 63.5 | 0.81 |
| Total cholesterol, mg/dL | 206 ± 49 | 207 ± 51 | 206 ± 47 | 0.89 |
| LDL cholesterol, mg/dL | 111 ± 43 | 117 ± 42 | 99 ± 42 | 0.12 |
| HDL cholesterol, mg/dL | 56 ± 17 | 55 ± 17 | 57 ± 16 | 0.38 |
| Triglycerides, mg/dL | 133 (97-195) | 132 (97-195) | 133 (91-190) | 0.73 |
| Lipoprotein(a), mg/dL | 15.2 (6.6-52.2) | 9.5 (3.9-15.2) | 62.0 (52.1-80.3) | <0.001 |
| OxPL-apoB, nM | 1.8 (1.2-4.8) | 1.3 (0.9-1.8) | 5.9 (4.7-9.2) | - |
| ***Medication*** |  |  |  |  |
| Statin at baseline | 47 (32.4) | 27 (27.8) | 20 (41.7) | 0.09 |
| Statin during follow-up | 82 (56.6) | 52 (53.6) | 30 (62.5) | 0.31 |
| ACE inhibitor | 43 (29.7) | 26 (26.8) | 17 (36.2) | 0.25 |
| ***Echocardiography*** |  |  |  |  |
| Peak aortic jet velocity, m/s | 3.42 ± 0.79 | 3.36 ± 0.74 | 3.56 ± 0.89 | 0.15 |
| Peak aortic valve gradient, mmHg | 48.9 ± 23.4 | 46.7 ± 20.5 | 53.4 ± 28.2 | 0.10 |
| Mean aortic valve gradient, mmHg | 26.7 ± 14.2 | 25.5 ± 12.7 | 29.2 ± 16.6 | 0.14 |
| Aortic valve area, cm2 | 1.07 ± 0.40 | 1.09 ± 0.42 | 1.04 ± 0.56 | 0.50 |

Data are presented as mean ± standard deviation, median (interquartile range, or number (percentage). LDL cholesterol was corrected for cholesterol content in Lp(a): LDL-c = LDL-c – Lp(a) mass * 0.3. OxPL-apoB = oxidized phospholipids on apolipoprotein B-100

**Supplemental Table 5. Multiple linear regression analysis (OxPL-apoB)**

| **Multiple linear regression models** | **18F-NaF PET activity: TBR_MDSmean_** | | | | **Progression in AV calcium score: ∆Ca-score (100 AU/yr)** | | | | **Change in peak aortic jet velocity: V_max, m/s/yr_** | | | |
| --- | --- | --- | --- | --- | --- | --- | --- | --- | --- | --- | --- | --- |
| **Variable** | **Unstd. β (95% CI)** | **Std. β** | **p-value** | **Unst. β (95% CI)** | | **Std. β** | **p-value** | **Unstd. β (95% CI)** | | **Std. β** | **p-value** |  |
| Age, per 10 years | 0.091 (-0.033 to 0.215) | 0.171 | 0.147 | -0.282 (-1.052 to 0.489) | | -0.090 | 0.464 | 0.007 (-0.033 to 0.048) | | 0.036 | 0.713 |  |
| Male sex | 0.167 (-0.094 to 0.428) | 0.192 | 0.206 | 1.094 (-0.437 to 2.624) | | 0.236 | 0.156 | -0.031 (-0.122 to 0.059) | | -0.070 | 0.494 |  |
| BMI, kg/m^2^ | 0.023 (0.000 to 0.046) | 0.228 | 0.052 | -0.083 (-0.272 to 0.107) | | -0.122 | 0.383 | 0.004 (-0.005 to 0.014) | | 0.002 | 0.355 |  |
| Cardiovascular disease | 0.050 (-0.170 to 0.270) | 0.059 | 0.652 | 0.281 (-0.911 to 1.472) | | 0.064 | 0.636 | 0.027 (-0.058 to 0.112) | | 0.064 | 0.533 |  |
| Smoking | -0.031 (-0.199 to 0.136) | -0.038 | 0.708 | -0.433 (-1.370 to 0.504) | | -0.098 | 0.356 | -0.014 (-0.094 to 0.065) | | -0.034 | 0.723 |  |
| Diabetes | -0.215 (-0.479 to 0.049) | -0.193 | 0.108 | -0.328 (-1.968 to 1.311) | | 0.010 | 0.948 | -0.084 (-0.219 to 0.052) | | -0.121 | 0.223 |  |
| Hypertension | 0.062 (-0.140 to 0.264) | 0.068 | 0.541 | -0.368 (-1.534 to 0.798) | | -0.075 | 0.527 | -0.029 (-0.109 to 0.050) | | -0.072 | 0.464 |  |
| Creatinine, per 10 mg/dL | -0.002 (-0.033 to 0.029) | -0.017 | 0.881 | -0.237 (-0.441 to -0.032) | | -0.299 | 0.024 | -0.002 (-0.018 to 0.015) | | -0.019 | 0.850 |  |
| OxPL-apoB top tertile | 0.204 (0.040 to 0.367) | 0.244 | 0.016 | 1.365 (0.426 to 2.304) | | 0.305 | 0.005 | 0.080 (-0.002 to 0.161) | | 0.179 | 0.054 |  |
| Baseline AV Ca-score, per 100 AU | 0.013 (0.007 to 0.018) | 0.528 | <0.001 | 0.111 (0.073 to 0.150) | | 0.675 | <0.001 | - | | - | - |  |
| Baseline V_max_, m/s | - | - | - | - | | - | - | 0.041 (-0.011 to 0.093) | | 0.147 | 0.120 |  |

BMI = body mass index; AV Ca-score = aortic valve calcium score; AU = Agatston Units; V_max_ = peak aortic jet velocity; TBR_MDSmean_ = tissue-to-background ratio of the most diseased segment; Unst. β = unstandardized beta coefficient; Std. β = standardized beta coefficient; CI = confidence interval

**Supplemental Table 6. ATX-Lp(a) and ATX-apoB associations with valvular uptake of 18F-NaF, progression in CT calcium scoring and hemodynamic progression**

In an exploratory analysis, we assessed whether plasma levels of autotaxin (ATX) carried by Lp(a) and OxPL would improve stratification beyond Lp(a) and OxPL levels. ATX has recently been demonstrated to be a mediator of AS disease progression through the generation of lysophosphatidic acid.(2,5-7)

|  | **ATX-Lp(a)** | | | **ATX-apoB** | | |
| --- | --- | --- | --- | --- | --- | --- |
|  | Below median ≤ 2652 RLU | Above median > 2652 RLU | p-value | Below median ≤ 9310 RLU | Above median > 9310 RLU | p-value |
| **18F-NaF PET (TBR_MDSmean_)** | | | | | | |
| Total group (n=76) | 2.03±0.45 | 2.04±0.39 | 0.937 | 2.13±0.42 | 2.00±0.41 | 0.286 |
| Lp(a) tertile 1 & 2 | 2.02±0.54 | 1.93±0.39 | 0.530 | 2.14±0.50 | 1.93±0.44 | 0.195 |
| Lp(a) tertile 3 | 2.05±0.29 | 2.23±0.33 | 0.130 | 2.11±0.33 | 2.14±0.32 | 0.858 |
| OxPL-apoB tertile 1 & 2 | 2.14±0.50 | 1.92±0.44 | 0.195 | 2.14±0.50 | 1.93±0.44 | 0.216 |
| OxPL-apoB tertile 3 | 2.11±0.33 | 2.14±0.32 | 0.807 | 2.11±0.33 | 2.12±0.33 | 0.935 |
| **Baseline AV Ca-score (AU)** | | | | | | |
| Total group (n=68) | 1939±1834 | 1854±1711 | 0.843 | 2053±2099 | 1839±1646 | 0.668 |
| Lp(a) tertile 1 & 2 | 2181±1999 | 1767±1859 | 0.493 | 2082±2305 | 1901±1806 | 0.797 |
| Lp(a) tertile 3 | 1605±1594 | 2014±1455 | 0.502 | 2011±1942 | 1735±1374 | 0.689 |
| OxPL-apoB tertile 1 & 2 | 2181±1999 | 1763±1900 | 0.499 | 2082±2305 | 1903±1836 | 0.802 |
| OxPL-apoB tertile 3 | 1605±1594 | 2002±1399 | 0.497 | 2011±1942 | 1741±1338 | 0.687 |
| **ΔCa-score (AU/yr)** | | | | | | |
| Total group (n=48) | 226±200 | 245±210 | 0.744 | 284±177 | 217±213 | 0.301 |
| Lp(a) tertile 1 & 2 | 160±181 | 223±237 | 0.443 | 235±198 | 184±224 | 0.574 |
| Lp(a) tertile 3 | 305±200 | 297±131 | 0.919 | 350±131 | 277±184 | 0.407 |
| OxPL-apoB tertile 1 & 2 | 160±182 | 223±237 | 0.443 | 235±198 | 184±224 | 0.574 |
| OxPL-apoB tertile 3 | 305±200 | 297±131 | 0.919 | 350±131 | 277±184 | 0.407 |
| **Baseline V_max_ (m/s)** | | | | | | |
| Total group (n=141) | 3.43±0.80 | 3.43±0.79 | 0.983 | 3.40±0.74 | 3.46±0.84 | 0.650 |
| Lp(a) tertile 1 & 2 | 3.45±0.80 | 3.19±0.59 | 0.088 | 3.37±0.69 | 3.27±0.73 | 0.478 |
| Lp(a) tertile 3 | 3.33±0.74 | 3.73±0.93 | 0.152 | 3.42±0.82 | 3.61±0.89 | 0.488 |
| OxPL-apoB tertile 1 & 2 | 3.47±0.78 | 3.29±0.70 | 0.255 | 3.39±0.67 | 3.36±0.81 | 0.838 |
| OxPL-apoB tertile 3 | 3.36±0.83 | 3.77±0.92 | 0.128 | 3.42±0.88 | 3.69±0.89 | 0.310 |
| **ΔVmax (m/s/yr)** | | | | | | |
| Total group (n=126) | 0.14±0.21 | 0.21±0.19 | 0.066 | 0.21±0.21 | 0.14±0.19 | 0.050 |
| Lp(a) tertile 1 & 2 | 0.09±0.19 | 0.20±0.19 | 0.012 | 0.19±0.22 | 0.10±0.17 | 0.041 |
| Lp(a) tertile 3 | 0.23±0.23 | 0.24±0.18 | 0.810 | 0.24±0.21 | 0.23±0.21 | 0.812 |
| OxPL-apoB tertile 1 & 2 | 0.10±0.19 | 0.20±0.19 | 0.019 | 0.19±0.21 | 0.10±0.17 | 0.027 |
| OxPL-apoB tertile 3 | 0.23±0.23 | 0.24±0.19 | 0.836 | 0.24±0.22 | 0.23±0.21 | 0.874 |

AV Ca-score = aortic valve calcium score; AU = Agatston Units; V_max_ = peak aortic jet velocity; TBR_MDSmean_ = tissue-to-background ratio of the most diseased segment; RLU = relative light units.

| **Multiple linear regression model** | **Change in peak aortic jet velocity: V_max, (_m/s/yr)** | | |
| --- | --- | --- | --- |
| **Variable** | **Unstd. β (95% CI)** | **Std. β** | **p-value** |
| Model + ATX-Lp(a) above median | 0.084 (0.011-0.158) | 0.209 | 0.025 |
| Model + ATX-apoB above median | -0.069 (-0.145 to 0.007) | -0.171 | 0.075 |

Model is adjusted for Lp(a) top tertile (for ATX-Lp(a) or OxPL-apoB top tertile (for ATX-apoB), baseline peak aortic jet velocity and traditional cardiovascular risk factors (age, sex, body mass index, history of cardiovascular disease, smoking status, diabetes mellitus, hypertension and plasma creatinine).

**Supplemental Figure 1. Aortic valve replacement or cardiovascular death during follow-up (secondary clinical endpoint)**

Patients in the top (A) Lp(a) tertile and (B) OxPL-apoB tertile had more events than those in the lower tertiles. (C and D) Multivariate Cox proportional hazards analysis for aortic valve replacement surgery and death. Hazard ratio for age is per 10 years, BMI per kg/m2, creatinine per 10 mg/dL; V_max_ per m/s. BMI = body mass index; CVD = cardiovascular disease; V_max_ = peak aortic jet velocity; CI = confidence interval.


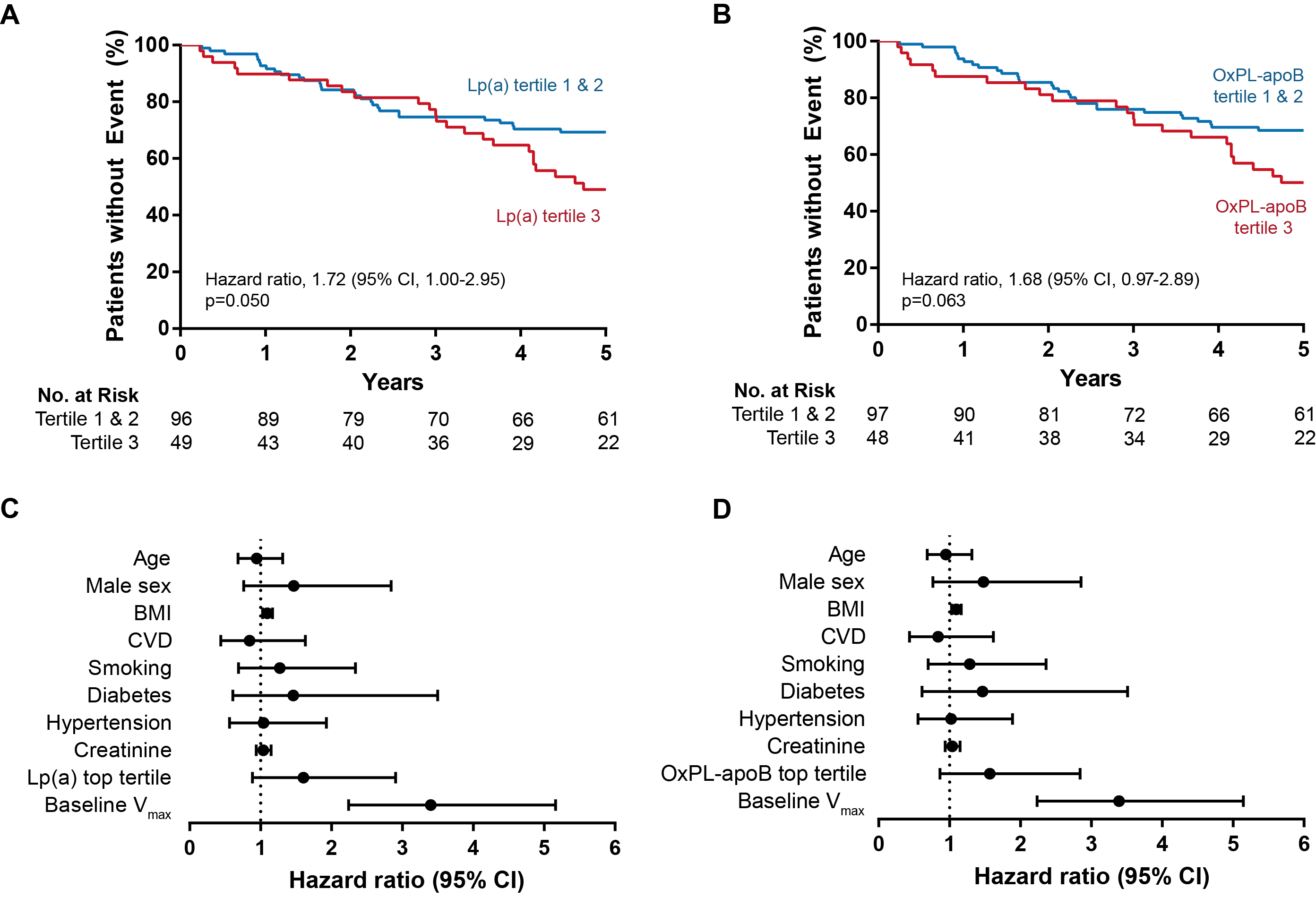


**Supplemental Figure 2. ATX-Lp(a) and ATX-apoB distribution and correlations with Lp(a) and OxPL-apoB**

(A) ATX-Lp(a) and (B) ATX-apoB distribution in the study cohort. There was no correlation between (C) ATX-Lp(a) and Lp(a) levels or (D) ATX-apoB and OxPL-apoB levels. RLU = relative light units.


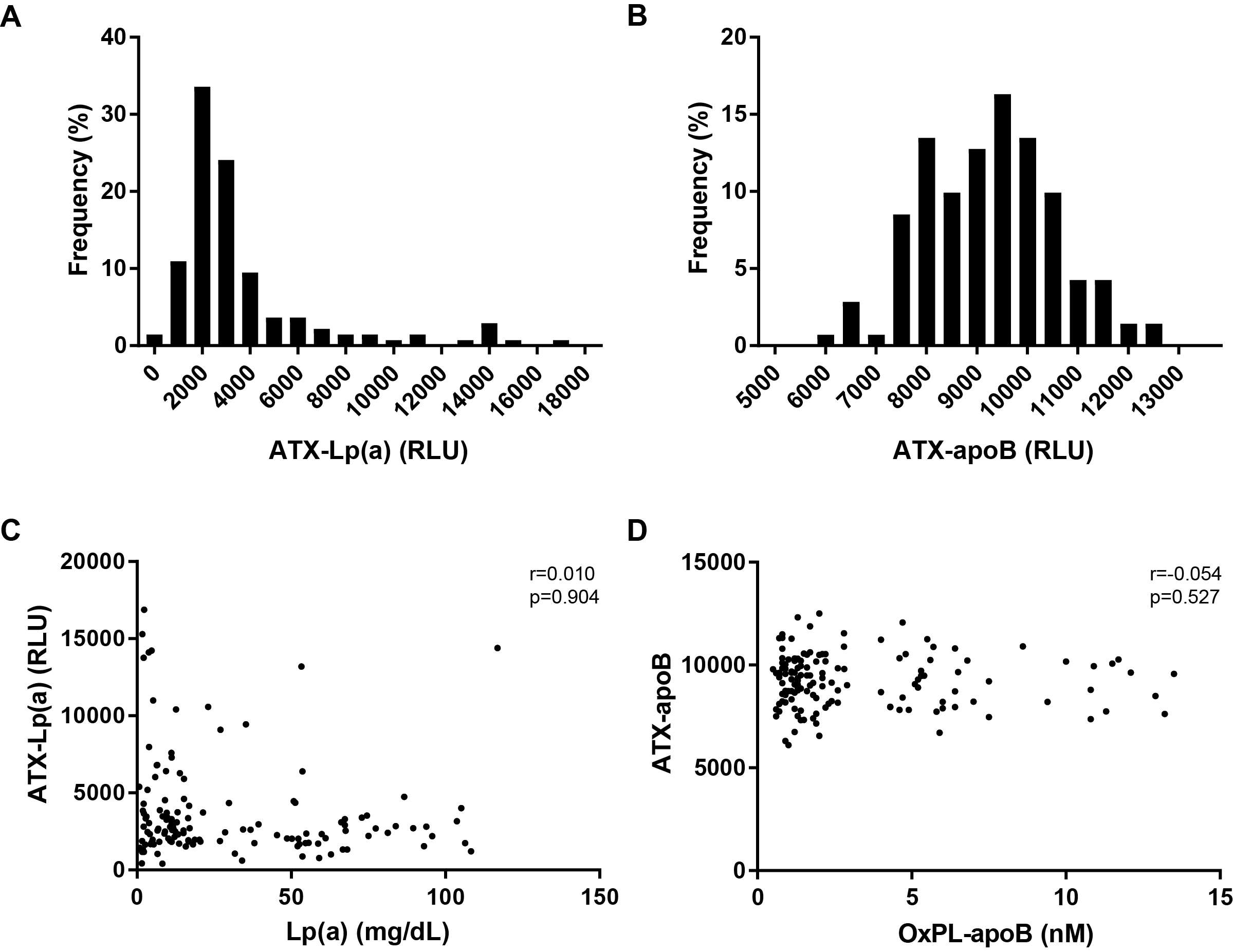


**Supplemental Figure 3. ATX-Lp(a) and ATX-apoB associations with AVR or death during follow-up**

(A+B) Kaplan-Meier curves demonstrate that there was no difference in event-free survival between patients with above median or below median levels of ATX-Lp(a) and ATX-apoB. Further stratification in (C+E) Lp(a) and (D+F) OxPL-apoB tertiles showed similar outcomes.

**
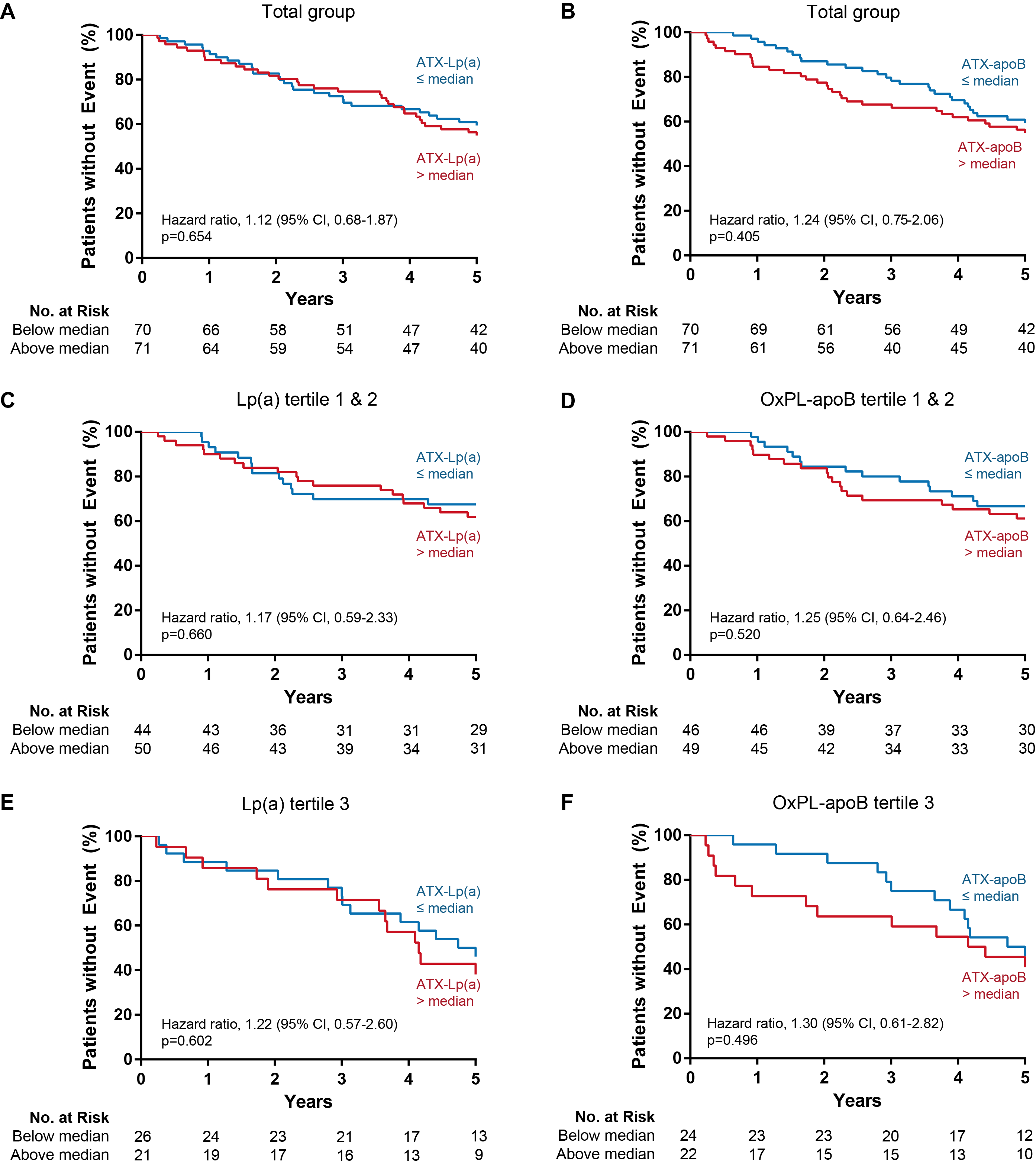
**

**Post hoc power analyses**

- Top versus lower OxPL-apoB tertiles on hemodynamic progression (0.23±0.21 m/s/year vs. 0.15±0.20 m/s/yr; p=0.054): post-hoc analysis revealed that a two group t-test with a two-sided significance level of 0.05, a total group size of n=129 and the observed effect size (δ=0.4; mean difference in V_max_ of 0.08 m/s/yr) had 55% power. The power was 74% to detect a moderately larger effect (δ=0.5; mean difference in V_max_ of 0.10 m/s/yr), whereas it was 88% to detect a large effect (δ=0.6; mean difference in V_max_ of 0.12 m/s/yr).
- Top versus lower OxPL-apoB tertiles for the secondary composite endpoint (23 [47.9%] vs. 30 [30.9%]; hazard ratio for top OxPL-apoB tertile: 1.68; 95% CI, 0.97 to 2.89, p=0.063): post-hoc analysis, on the basis of the group sizes of n=48 and n=97, revealed that a log-rank test for equality of survival curves with a 0.05 two-sided significance level had approximately 52% power to detect the difference between the observed survival curves. Statistical power was 81% to detect a larger effect size of 25% difference in incidence rate between the groups.

**References**

1. American College of Cardiology/American Heart Association Task Force on Practice G, Society of Cardiovascular A, Society for Cardiovascular A et al. ACC/AHA 2006 guidelines for the management of patients with valvular heart disease: a report of the American College of Cardiology/American Heart Association Task Force on Practice Guidelines (writing committee to revise the 1998 Guidelines for the Management of Patients With Valvular Heart Disease): developed in collaboration with the Society of Cardiovascular Anesthesiologists: endorsed by the Society for Cardiovascular Angiography and Interventions and the Society of Thoracic Surgeons. Circulation 2006;114:e84-231.

2. Bouchareb R, Mahmut A, Nsaibia MJ et al. Autotaxin Derived From Lipoprotein(a) and Valve Interstitial Cells Promotes Inflammation and Mineralization of the Aortic Valve. Circulation 2015;132:677-90.

3. van der Valk FM, Bekkering S, Kroon J et al. Oxidized Phospholipids on Lipoprotein(a) Elicit Arterial Wall Inflammation and an Inflammatory Monocyte Response in Humans. Circulation 2016;134:611-24.

4. Leibundgut G, Scipione C, Yin H et al. Determinants of binding of oxidized phospholipids on apolipoprotein (a) and lipoprotein (a). J Lipid Res 2013;54:2815-30.

5. Nsaibia MJ, Boulanger MC, Bouchareb R et al. OxLDL-derived lysophosphatidic acid promotes the progression of aortic valve stenosis through a LPAR1-RhoA-NF-kappaB pathway. Cardiovasc Res 2017;113:1351-1363.

6. Nsaibia MJ, Mahmut A, Boulanger MC et al. Autotaxin interacts with lipoprotein(a) and oxidized phospholipids in predicting the risk of calcific aortic valve stenosis in patients with coronary artery disease. J Intern Med 2016;280:509-517.

7. Torzewski M, Ravandi A, Yeang C et al. Lipoprotein(a)-Associated Molecules Are Prominent Components in Plasma and Valve Leaflets in Calcific Aortic Valve Stenosis. JACC: Basic to Translational Science 2017;2:229-240.
